# Unilateral Ureteric Endometriosis at the Pelvic Brim Resulting in Complete Loss of Renal Function

**DOI:** 10.1155/2019/9194615

**Published:** 2019-10-13

**Authors:** Keisuke Tanaka, Akwasi A. Amoako, Katherine Gray, David Baartz

**Affiliations:** ^1^Women's and Newborn Services, Royal Brisbane and Women's Hospital, Herston, Australia; ^2^Faculty of Medicine, The University of Queensland, Herston, Australia; ^3^Department of Urology, Royal Brisbane and Women's Hospital, Herston, Australia

## Abstract

Deep infiltrating endometriosis of the urinary tract is rare but can result in ureteric obstruction, hydroureteronephrosis and renal failure. Ureteric endometriosis usually affects the distal third of the left ureter among women of reproductive age. Greater awareness of ureteric endometriosis and a multidisciplinary approach in the management is essential to achieve optimal outcomes. We present an atypical case of right ureteric obstruction due to endometriosis at the pelvic brim resulting in complete loss of renal function and necessitating nephroureterectomy.

## 1. Introduction

Endometriosis affects 10%–15% of all women of reproductive age [1]. Women often present with symptoms such as dysmenorrhoea, pelvic pain, dyspareunia, bowel upset (e.g. constipation, diarrhoea) or pain and infertility [2]. There are 3 major phenotypes of endometriosis: ovarian endometrioma, superficial peritoneal endometriosis, and deep infiltrating endometriosis (DIE) [3]. DIE is defined as endometriosis infiltrating the peritoneum deeper than 5 mm [4] and is recognised as the most aggressive form of endometriosis. DIE of the urinary system is rare and is found in about 1%–5.5% of all women with endometriosis [5], with the bladder, ureter, and kidney being affected in a ratio of 40 : 5 : 1 [6].

We present a case of complete loss of one kidney function secondary to ureteric endometriosis at the pelvic brim causing hydroureteronephrosis requiring laparoscopic unilateral nephroureterectomy.

## 2. Case Presentation

**Figure 1:**
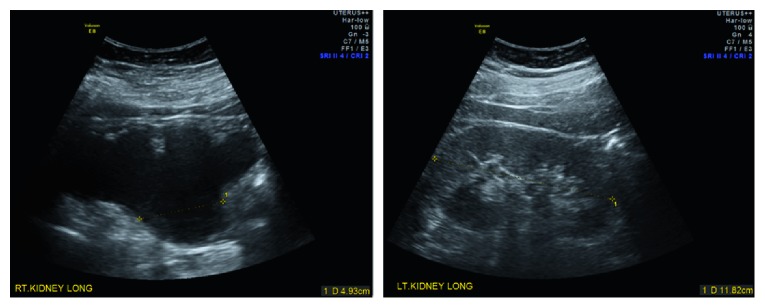
Kidney ultrasound scan showed severe right hydronephrosis with parenchymal thinning and normal left kidney.

**Figure 2:**
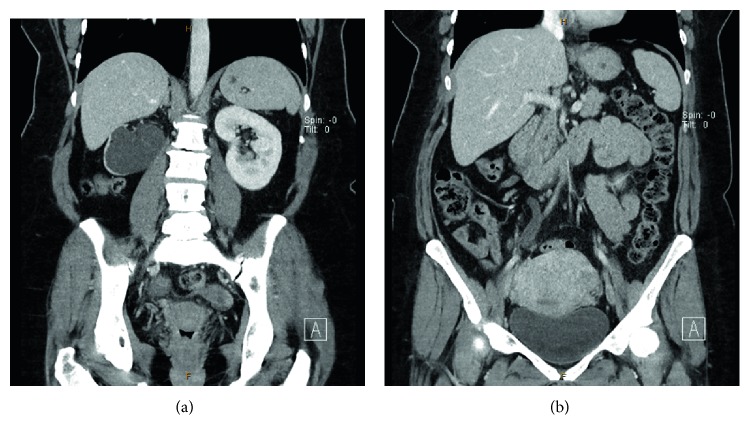
CT urogram confirmed significant right hydronephrosis with marked thinning of the renal parenchyma (a), and right hydroureter at the level of the pelvic brim where it crosses the common iliac artery (b).

A 47-year-old female, gravida 2 para 0, presented to a general practitioner with fatigue. She had no significant medical or surgical history. Her menstrual cycle was regular and not painful, and she had never seen a gynaecologist. A series of investigations were organised, including a pelvic ultrasound scan which revealed an enlarged, adenomyotic uterus (84 × 67 × 88 mm, volume 260 cc). Both ovaries were normal. An ultrasound scan of the upper renal tract incidentally noted significant right hydronephrosis, with a renal pelvis anterior-posterior diameter of 42 mm and the presence of parenchymal thinning (Figure 1). A CT urogram confirmed the significant right hydronephrosis with marked thinning of the renal parenchyma (Figure 2). On delayed imaging, there was minimal contrast uptake by the affected kidney suggesting no effective excretion. There was no renal, ureteric or bladder calculi seen. The dilated right ureter was identified at the pelvic brim, with surrounding soft tissue density. She was then referred to urology. Blood tests showed CA 125 of 44 U/mL (<30), CA 19.9 of 29 U/mL (<30), and CEA < 0.5 ug/L (up to 4.6). Renal function test was normal. Urea was 5.5 mmol/L (2.5–7.0), Creatinine was 72 mmol/L (45–85), and eGFR was 86 (>59).

**Figure 3:**
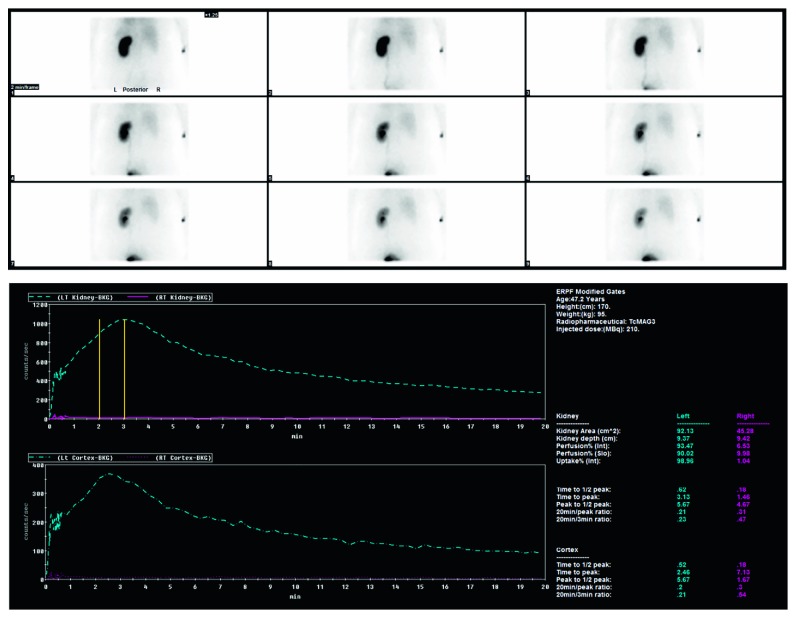
MAG3 scintigraphy showing complete loss of function of the right kidney.

**Figure 4:**
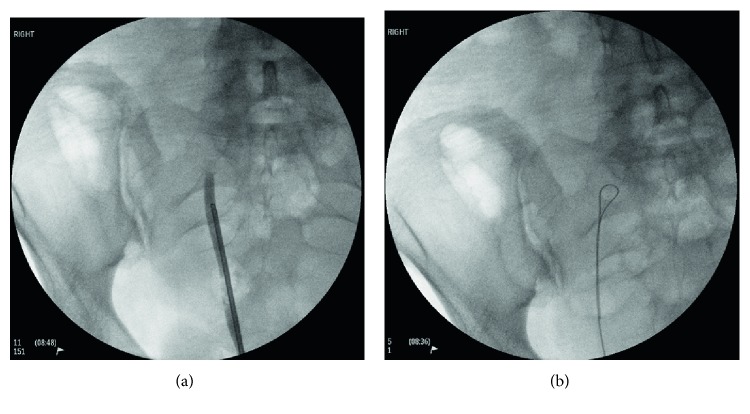
Right retrograde pyelogram showing a blind-ending right ureter at the level of the pelvic brim (a), where a wire could not be passed through the obstruction (b).

**Figure 5:**
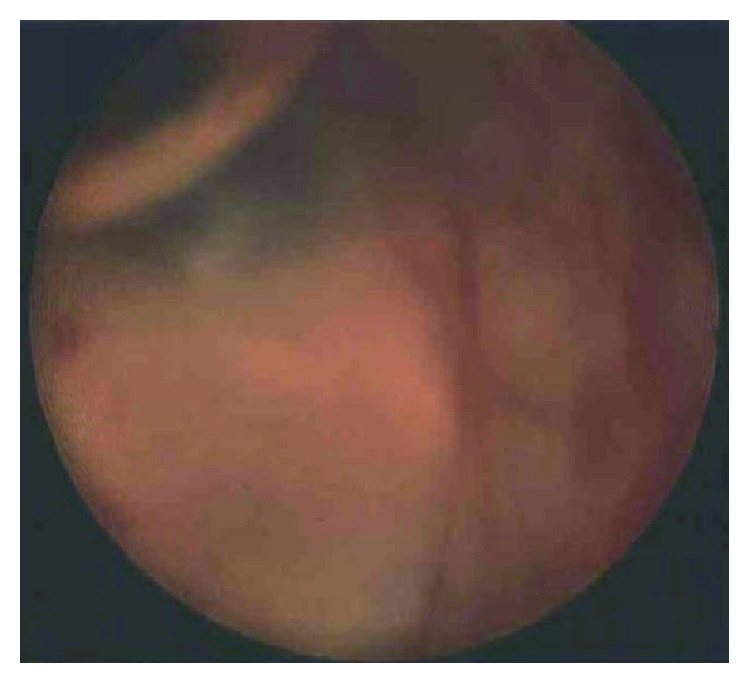
Ureteroscopy showing a blind-ending right ureter compressed by a mass with purple discolouration at the level of the pelvic brim.

Urine cytology showed red blood cells but it was negative for high-grade urothelial carcinoma. A MAG 3 scintigraphy showed that the right kidney was nonfunctional (Figure 3). Cystoscopy, right retrograde pyelogram (Figure 4) and ureteroscopy (Figure 5) showed a normal bladder, and a blind-ending right ureter compressed by a mass with purple discolouration at the level of the pelvic brim. There was no evidence of mucosal abnormality, but a wire could not be passed through the obstruction. She was referred to gynaecology with a likely diagnosis of endometriosis.

**Figure 6:**
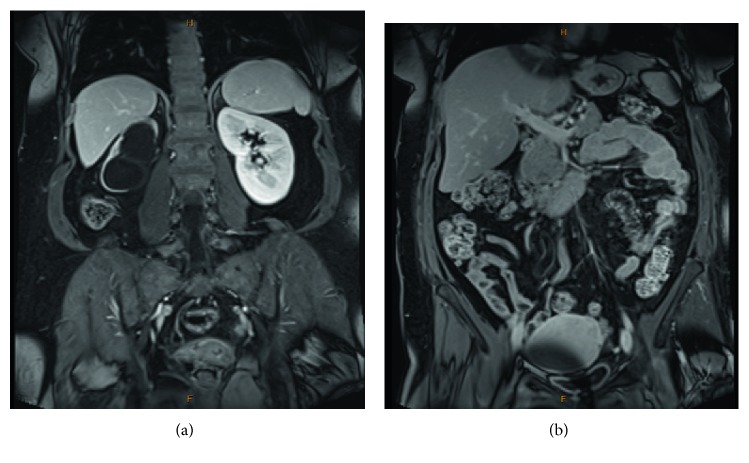
MRI confirmed significant right hydronephrosis with marked thinning of the renal parenchyma (a), and right hydroureter (b).

An MRI confirmed the presence of gross right-sided hydronephrosis and hydroureter to the level of the pelvic brim, and the ureter was not visible below this point (Figure 6). Marked thinning of the right renal cortex was noted. There was no mass seen adjacent to the ureter at the pelvic brim, however several small bowel loops lied in close proximity.

**Figure 7:**
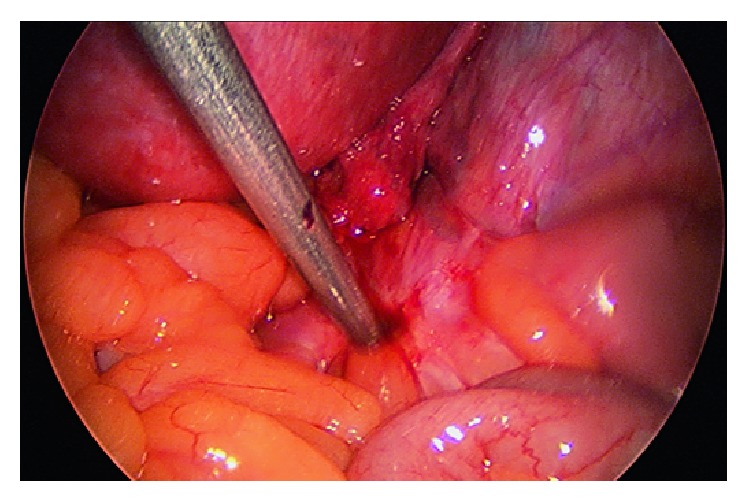
Laparoscopy revealed a dense 3 cm nodule over the right ureter at the pelvic brim involving the small bowel mesentery.

Severe endometriosis was suspected based on the findings of unilateral complete loss of renal function due to ureteric obstruction and the presence of adenomyosis. Patient did not consent for definitive surgery, largely due to the fact that she was asymptomatic, her quality of life was not affected, and her right renal function had been lost completely already. Decision was made for a laparoscopy to confirm the diagnosis of endometriosis and to assess the severity of the disease in order to facilitate more detailed discussion of required procedure and its risks before proceeding further. Laparoscopy revealed a dense 3 cm nodule over the right ureter at the pelvic brim involving the small bowel mesentery (Figure 7). The uterus was bulky and the rectum was adherent to the posterior uterine wall. Both ovaries were adherent to the pelvic side wall, and a small endometrioma was drained from the right ovary. Definitive surgery would have involved extensive dissection and adhesiolysis to normalise the pelvic anatomy, ureterolysis, hysterectomy, bilateral salpingo-oophrectomy, excision of endometriosis possibly involving the bowel, and right nephroureterectomy performed by a multidisciplinary team. Patient decided not to proceed with such definitive surgery as she was not prepared to take the surgical risks particularly relating to possible bowel resection. Instead, she was commenced on progesterone therapy for endometriosis and underwent laparoscopic transperitoneal right nephroureterectomy to prevent pyonephrosis in the future. A decision was made to only remove the kidney and upper ureter rather than removal of the ureter in its entirety to the vesico-ureteric junction to minimise the morbidity from this procedure. The kidney was dissected free from its attachments, drained of urine and removed via a laparoscopic port site. The procedure was uncomplicated.

16 months post nephroureterectomy, at the age of 49, she remained asymptomatic of endometriosis but reported ongoing fatigue. A recent ultrasound showed normal left urinary tract without signs of hydronephrosis, and a MAG 3 scintigraphy showed normal perfusion, function and drainage of the left kidney. She is to remain on progesterone therapy until menopause and to have regular pelvic ultrasounds to ensure the left urinary tract is not affected.

## 3. Discussion

Many aspects of our case are atypical of ureteric endometriosis, including age, symptomatology, the side of ureteric endometriosis, level of ureteric obstruction, and the degree of renal dysfunction. Three large studies with 96 or more women with ureteric endometriosis reported a mean age between 34 and 36.1 years respectively [7–9]. Our patient was of an older age of 47 years old, and this was an important factor in considering her treatment options. She would have reached the average age of menopause within a few years, and there was no obstruction in the left ureter. Therefore she was more suitable for conservative management with medical therapy than the younger cohorts.

Women with ureteric endometriosis are usually symptomatic. Dysmenorrhoea, pelvic pain, dyspareunia, and dyschezia were reported in 70.6%–83%, 36.8%–52.3%, 33.0%–47.5%, and 20.8%–47.5% of affected women respectively [5, 7–9]. The only symptom in our case was fatigue. This inevitably led to delay in seeking medical advice resulting in complete loss of renal function in one kidney. Obstructive kidney injury due to ureteric endometriosis has been reported [7–13], though complete loss of renal function in one kidney is rare [14].

Obstructing ureteric endometriosis are commonly found on the left-side at the level of the ovarian fossa or in correspondence with the ureterosacral ligament [15]. Left-sided dominance supports the hypothesis of retrograde menstruation as a cause of endometriosis [16]. The presence of the rectosigmoid on the left provides an anatomical shelter that may protect endometriotic cells from being cleared by both the peritoneal circulation and macrophages, thus favouring their peritoneal adhesion and subsequent deep penetration [17]. In our case, the endometriotic nodule was found to compress the right ureter against the pelvic brim. It is worth noting that the pathophysiology of endometriosis remains elusive, and other proposed theories include coelomic metaplasia, residual embryonic Mullerian rests, benign metastasis via the lymphatic or haematological circulation, and genetic factors [18].

It can be challenging to reach a correct diagnosis for ureteric obstruction, as there are a number of differential diagnoses. Noncongenital causes include (1) urolithiasis, (2) primary carcinoma of the ureter, (3) nonurologic malignancies including ovarian, cervical, colorectal, breast and lymphoma, (4) inflammatory due to surgery, trauma, radiotherapy, endometriosis, infections, abdominal aortic aneurysm, retroperitoneal fibrosis or idiopathically, and (5) pregnancy [19]. However some initial investigation results were suggestive of endometriosis in our case. The pelvic ultrasound scan showed adenomyosis, a condition closely related to endometriosis, as 79% of women with endometriosis were found to have adenomyotic lesions [20]. Similarly, 42.3% of women with histologically proven adenomyosis were also diagnosed with endometriosis [21]. Furthermore, serologic testing for CA 125 was elevated at 44 U/mL, and this marker has been used widely not only for epithelial ovarian cancer, but also for detection of endometriosis. One study reported a positive predictive value of 92.9% at the cut off value of 35 U/mL in the diagnosis of endometriosis without endometriomas [22]. In our case the right kidney function was already lost at the time of diagnosis and therefore could not have been salvaged. However it is important for women with ureteric obstruction to be seen by urology as soon as possible, as renal function may improve after urinary diversions with ureteric stenting or percutaneous nephrostomy.

Medical management alone is contraindicated in the setting of ureteral obstruction and hydronephrosis, and is not ideal for any ureteric endometriosis given the increased risk of recurrence and renal impairment [18]. The aim of surgical therapy for ureteric endometriosis is to relieve the obstruction, rescuing the involved kidney if possible, while minimising the risk of recurrence, and the choice of technique depends on the ureteric extension of the endometriosis and on renal function [18, 23, 24]. Ureterolysis is suitable for patients with no or mild obstruction. Ureteric endometriosis had been successfully removed by laparoscopic ureterolysis in 95% of cases (76 out of 80), whereas further surgery was needed for 5% of patients (4 out of 80) with more severe ureteric stenosis in a prospective study [25]. Ureterectomy with end-to-end anastomosis may be indicated for patients with severe or complete ureteral obstruction affecting a limited portion in the upper two thirds of the ureteral tract [16]. Ureterectomy with end-to-end anastomosis had been successful in 88% of cases (15 out of 17 women), though the other two cases required ureteroneocystostomy for persistent ureteral stenosis [26]. Patients with moderate to severe obstruction localised to the lower third of the ureter may require ureteroneocystostomy, whereas nephroureterectomy is indicated when a small portion of the kidney remains functional (less than 10%–15% on scintigraphy) and recovery of renal function is unlikely [24].

There are 3 general categories for surgical management for bowel endometriosis: shaving excision, disc resection, and segmental resection, and the choice depends on the location of the bowel lesion, depth of infiltration, number of nodules, and presence or absence of stricture [27]. There is a growing number of evidence to suggest less invasive options as associated with favourable symptom relief, fewer complication rate, but with some risk of failure, recurrence and need for further surgery [28–30]. It is possible that our patient would have been suitable for less invasive options based on assessment after pelvic dissection and adhesiolysis, nonetheless she needed to be consented for possible segmental resection.

Patient's age, symptomatology and preference eventually dictated the management plan in our case. Although medical therapy alone is usually not recommended in the setting of ureteric endometriosis, her renal function had been lost completely on one side at the time of diagnosis and the other ureter was not involved. She was asymptomatic and thought to have a few years before reaching menopause, limiting opportunity for endometriosis to affect and obstruct the remaining functioning ureter. Therefore, we believe, not proceeding with the definitive surgery and avoiding surgical risks while respecting patient's autonomy was a reasonable option.

Our case illustrated the challenge of reaching a diagnosis of ureteric endometriosis due to its rare incidence and nonspecific symptomatology. With a rare but catastrophic consequence of losing a kidney, a greater awareness of ureteric endometriosis among specialities including general practitioners, urologists, colorectal surgeons and gynaecologists is needed. We recommend considering pelvic ultrasound scan and CA125 blood test as a part of the initial investigations for women of reproductive age even in the setting where symptomatology is nonspecific and not suggestive of endometriosis.
